# Divinity of the Law

**Published:** 1895-01

**Authors:** J. H. Allen

**Affiliations:** Logansport, Ind.


### DIVINITY OF THE LAW.

J. H. ALLEN, M. D., LOGANSPORT, IND.

With the Genesis of creation comes the Genesis of law. Physical law and the physical universe are inseparable. You cannot study one without the other. The smallest atom of matter is under the government of the same forces—under the influence of the same laws as the planets. Cohesion is in every known thing. Gravitation is everywhere, bringing to bear its influences in harmony. God's laws are His commands executed;

His will evolving itself; the source of which is in His omnipotence. They are the dictates to nature, whether revealed or unrevealed. They are the decrees of Heaven. The universe and all creation is subject to them. Chaos would return, and the mind cannot imagine the results if the physical laws of our universe were annulled. In this we may consider not only the inorganic, but the organic. Life itself is but the outgrowth, and the development of law. "From harmony, from heavenly harmony, this universal world began," through the potency and omnipresence of law. It may be considered the priora of matter, for without law matter could not exist. It transcended and evolved from the forces through the divine instrumentality of law; through the conservation, correlation, and transference of forces.

The same physical laws prevail everywhere and in everything. It is the same law that forms an iceberg in the Arctic Seas as that which forms the Queen of Crystals in the mine; and by the aid of chemical analysis, the telescope, the microscope, and the spectroscope, we find that all parts of this universe are constructed on the same grand plan. The same light that revealed creation shines through everything. It is the same light that paints the sunbeam, that lights the insect's wing, the diamond's flash, or the water's crystal drop, as that which is revealed in the spectroscope.

Though natural law predicates nothing, of course, it is a statement of the orderly condition of things in nature. Drummond says of natural laws, that "They are great lines running, not only through this world, but through the universe, reducing it in parallels of latitude to intelligent order."

The discovery of law is the discovery of science. Law undertakes the profound task of comparing line by line, angle by angle, and point by point. If natural law could be traced to its spiritual source it would bring with it all the credentials that the thinking mind requires. The effects of the introduction of natural laws among the recorded phenomena of nature has simply made science. It has transformed knowledge into truth; it has driven away mysticism; cleared from the mind the shadows of doubt and superstition, and brought us into a closer relationship with the Divine Author of all that is good and perfect.

When the mind grasps these natural or physical laws a revelation begins, and we come forth into the light of knowledge upon a broader basis; abandoning the negative side of things, we stand upon the broader platform of the positive. We see the truth in nature as it comes from the source of truth, and no one can study these laws without a change coming over his views of truth, as he grasps the actual things that are revealed to him through the knowledge of the law, his past knowledge, which has been studied from the standpoint of empiricism, becomes unfixed and unstable and he gropes about no longer in the darkness of uncertainty, but if we understand the laws of a science the phenomena will arrange themselves as we progress in the knowledge of them. New worlds may be discovered or new elements be brought to light, but the old laws of nature are found to govern them.

We, as children of nature, and being so limited in our vision, catch with the camera of the mind but a faint outline of the true picture of the outwardly expressed phenomena of these natural laws.

A limited number of our sciences may be designated as true sciences, but the majority are studied from the standpoint of a working hypothesis. Hudson, in his *Law of Psychic Phenomena*, says: "Substantial progress in a science is impossible in the absence of a working hypothesis, which is universal in its application to the phenomena pertaining to the subject-matter." It ceases to be an hypothesis as soon as it is demonstrated to be a law. It was a law unrevealed by demonstration, yet not knowing it, we call it an hypothesis until we find it controls and governs the mass of the phenomena pertaining to that science; therefore, no proposition in any science is worthy of belief unless demonstrated or verified by the phenomena. We may lose sight of a substance, but we cannot of a law. Its reign comprises the things invisible as well as the visible.

Law stops not with potency, and though it is hermetically sealed it is approachable from the natural side. "No key from the mineral world can open the door between the organic and the inorganic or from the vegetable to the animal nor from the natural to the spiritual" except through our knowledge of law. Spiritual laws are not only analogous to the natural, but they are the same. It is a question of identity. "The laws of the visible are not projections into the invisible, but a continuation." Therefore, as long as we are in the natural sphere we familiarize ourselves with nature through and by natural laws, but if we enter the sphere of the supernatural we do so through the power of the mind, evolving what we are not positive is outside of the natural through our acquaintance with law. What we to-day consider the supernatural to-morrow is classified among the natural, and what would appear a divine effort now is a human effort ten years hence. So the imaginary line between the natural and the supernatural is ever changing, as man's intelligence rises to a higher standard in his knowledge of truth. Do not we, as homœopathic physicians, perform cures every day that are in one sense of the word superhuman by the use of means applied in harmony with a natural law? and we as instruments of God on this esoteric plain cannot tell how far we may carry out His will through the divinity of His law.

As this is true of many of the sciences, it is especially true of the law of similia. The means employed are beyond human contrivance. Nature is sick, or, as Hahnemann has said, "There is a disturbance in these forces that maintain life," either from its natural environments or its unnatural, and the cure is more natural. Thus we bridge over these breaches in nature, whose cause is in sin, by the grace of God through the instrumentality of law. The law responds promptly as far as our intelligence or knowledge of the law make it possible for us to apply it.

Lew Wallace, in his *Prince of India*, says: "Similarity is a law of Nature, and the law of Nature is the will of God." Not only is the law divine, but the knowledge of it is divine; and when we are taught to look away from the tangible and to realize that that which is called spiritual is above the tangible, then we will begin to understand the beauties of pure Homœopathy and the potencies. Those who only believe in what they understand and take cognizance of by the five senses are bound as a slave to the material, and have not drunk from the fountain of enlightenment. They are dead, as far as their internal relationship is concerned, to a higher law and a more spiritual sight. An insight into pure Homœopathy is gained only by climbing higher than the materiality of things, and it is only through the understanding conception that we see the workings of the divine.

Our greatest discoverers of law have been guided to the opening of those paths by that delicate sense for abstract truth. Their minds were highly trained in the exercise of speculative thought; they almost guessed the truth before they proved the phenomena. Every true scientist must come to learn this fact, "that the things we see are not made of the things that do appear," and that the invisible forces lie behind and above all material things.

Since man became a dweller in this mundane sphere the law of similia has existed, and has spoken to us through the same phenomena as now, but we knew not the voice, and we might echo the words of the poet, "Science moves, but slowly, slowly, creeping up from point to point." The world was not yet ready for it. A man had to be born as a fit receptacle for such a mighty truth. When God wishes to carry out His will by conveying to the world some great truth, for the benefit of mankind, He creates a man; and as this is true of every great reform, it is especially true of Hahnemann. In him we find that Nature did so mix the elements as to bring forth a mind gifted with that power of thought that looks into the antecedents of things; gifted with a power of reasoning; an analytical mind; free from the narrowness and the prejudice of the scientists of his day. To such a man was revealed, through the limited knowledge then known, this wonderful natural law of *similia, similibus, curantur.*

Not only did Hahnemann discover a law of therapeutics, but he recognized a law of biogenesis that is in harmony with the law of similia. "Life," he says, "is a vital principle, a selfmoving force, a vital power, which, if acting in harmony preserves our bodies a harmonious whole; a disturbance of which is disease; a lack of which is death." True, it is a vital power; a means by which the biological forces are adjusted. As gravitation balánces all other forces in the physical world, so the life forces balance all other forces within the law of biology. This, of course, is in health.

Herbert Spencer says: "Life is a continuous adjustment of internal relations to external relations." It is by virtue of these correspondences that we are entitled to be called alive. We are constantly adjusting ourselves to change, so that any altered conditions in these life forces throw us out of correspondence with our environments. Any environment may be a cause of disease, and the effects be in proportion to our correspondence. If we correspond not with a part, the part is affected; with the whole, the whole is affected.

Besides the natural environments as the cause of disease we have a more prolific cause in what may be termed unnatural environments; certain poisonous influences known as miasmatic poisons or morbid forces that are antagonistic to life, or antagonistic to these forces that give us the manifestations of life.

Just why these morbid forces should exist we do not know. We do know, however, that they do exist and are in direct opposition and not in harmony with these healthy life forces in all organized life. It is impossible to trace the cause to its source. Hahnemann's teachings go deeper than the materiality of things. He recognizes a spiritual man dwelling within the natural man; both subject to change on account of their relationship. The vivifying principle he calls the *life force,* which, when acting normally, is in correspondence with all other forces, and in disease they are subject to their influences. When do they lose their correspondence? When they lose their internal relationship. When do these biological forces become subject to other forces? When the life forces have lost their resistive power by the presence of miasmatic forces from without. (Hahnemann—working hypothesis.) Both men, you see, have a similar conception of life, disease, and death. Both great minds modestly stop at the brink of the unknown shore: both recognize in life biological forces subject to biological law. In both cases the thought is an abstract one, yet they bring us into a closer relationship with the phenomena, and a better understanding of these natural laws. *Organon*, 10th paragraph: "Without this vital, dynamic power the organism is dead; by a disturbance of it the body is called sick;" the extent and intensity depending on the nature and intensity of the disturbance. It is then subject to the physical laws in proportion to the disturbance, or in other words, the biological forces that govern or control these healthy life forces are disturbed, leaving them more or less subject to the physical forces. Here Hahnemann's law of similia substitutes a similar force of nature from without, which acting on the disturbed life force within, restores it to a normal condition. Spencer could not disengage himself from the material. Hahnemann had long ago left the material plane, as he plainly saw that both life and disease were not material in their nature but dynamic or spirit-like. He had familiarized himself with the spiritual, which he knew to be above and before the material.

If we study not these laws of correspondence with relation to man and these physical laws, how can we expect to treat the man when out of correspondence with these laws, for they are the illuminators of nature, the dispensers of this gloom and darkness that surrounds us on every side. Within the organism lies the principles of life and it does not exist independent of antecedent life. In the environments are the conditions of life, and nature is its complement. We are only as we are sustained, and subsist only as we receive our environments. Health is perfect co-ordination; disease is the want of it. The proper simillimum restores this co-ordination, and as soon as it is restored health follows rapidly on the way. This explains the many instantaneous cures following the use of a high potency of the simillimum. They are so closely related to the co-ordinating power that they effect a cure quicker than the lower potencies, especially when there is great weakness in the recuperative power of the vital force.

The proper simillimum in order to restore this co-ordination must be not only akin to the life force but it must be a blood relation to it, as it were. It must have the like qualities and pecularities of the force that it is to correlate.

In the study of these divine laws of the force world we find that force cannot destroy force, nor can it neutralize it, but it can correlate it, or it can be changed from one kind of energy to another.

Disease appears and disappears by virtue of this same law. The activity of the drug energy will depend on its potentiality. The higher the potency the higher the energy, and *vice versa.*

Again, there is another feature of importance that must be taken into consideration in order to make a perfect application of the law so wonderfully brought out by Dr. Fincke; that is, the potentiality of the drug shall be equal to the potentiality of the life force. This potentiality will, of course, depend much on the susceptibility, or rather, the sensitivity of the organism. Therefore, the sensitivity must govern more or less the homœopathicity of the life force.

Ameke says: "None but the careful observer can have any idea of the height to which the sensitivness of the human body to medicines is increased in disease." It transcends all belief, especially where the disease has attained great intensity. There is no measure or weight to God's laws. He measures with a far-searching eye and weighs nature in the balance of the infinite.

The dynamis of the drug must be equal to the dynamis of the life force; so if the potentiality of the organism is far below the normal the potentiality of the drug may have to be changed so as to meet it, by selecting a lower or a higher potency, as the case may be. There is no rule, however, by which we may be guided to the proper potency, save that of experience and a study of the symptoms or the reaction of the life force.

Science is but what the natural world reveals to us through its phenomena, so the study of pure Homœopathy is but a study of its phenomena. We have for our consideration four sets of phenomena: the action of our remedies on the healthy; the study of their action upon the sick, or the reaction of the life force; also a study of the symptoms that disturb the life force, and the symptoms of the disturbed life force.

The study of these miasms that disturb the life force has been sadly neglected since Hahnemann's time. But a limited number to-day give them the proper consideration, which has greatly barred the progress of our science. *Organon*, paragraph 48, says: "The physician is likewise the guardian of health when he knows the objects that disturb it." If Hahnemann's chronic miasms are the *prima causa* of disease, how necessary it is for us to study them carefully. If we know not the symptoms by which they demonstrate their presence in the organism, how can we apply the law intelligently? The whole of our success depends on how we arrange the phenomena. Life is not a straight line, but a succession of curves and angles, and if we leave out one or more of these curves or angles in taking our case we have left out a part of the phenomena, and we cannot apply similia with certainty. The better we become acquainted with these natural laws, and these physical forces that retain or antagonize life, the better we can apply similia.

Hahnemann saw the divinity of the law of similia, and it was by virtue of his being in the spirit of truth that the law was revealed to him. *To him only who is a disciple of the master and has within him a desire for the truth will it unfold itself. It is by this spirit of truth that we are united, and it is the duty of all to go, if possible, farther into the research of this truth than did Hahnemann.* If it is divine it is infinite. If it is infinite it has no stopping-place. To Hahnemann it was an unknown and untrodden path stretching out into infinity. Shall we stop where Hahnemann stopped, or shall we go on in this path that grows no broader or no narrower, but onward and upward until the completion and fulfillment of the grace of Him who is the source of the divine?

Who among you needs further proof that disease is dynamic when you are all willing to testify that it can be cured by dynamics? Who, again, can say that disease can be local in its effects, knowing that this vital principle is an indivisible whole? And as "one touch of Nature makes the whole world kin," so what disturbs the organism in any part is felt in the whole. It is only a living part by virtue of the whole, or by virtue of the life force that vivifies the whole. Unless we accept the truth of a distributing force being inherent in disease we cannot accept it being inherent in drugs. Hence the close relationship between the disease and a potency of similia. It is fixed. Hahnemann has engraved it on the very door-posts of his temple of immortality; and we, as members of this association who worship at the shrine of this beautiful temple that God has given us by a revelation through this immortal Hahnemann, should approach its shrine with due reverence, knowing that only "he who sees takes off his shoes."

A Sandow may demonstrate to a phenomenal degree the power of those physical forces that are stored in his muscles, but a true scientific, homœopathic physician can demonstrate to the world in the cure of the sick a power that is from above through his acquaintance with the law of similia.

It stands as a guardian of health upon the threshold between life and death, offering a protecting and helping hand in all the ills that flesh is heir to. It soothes with gentle touch and unseen hand the very tempest of the mind; the aching and the fevered brow is cooled; our pains and our sufferings are by that potent touch dispelled. What a power lies within these potencies when applied through the law of similia! And whatever there is in life of any kind this law holds good. But we cannot materialize it, for it is a step above materialism; so we cannot discuss this science on a material plane, and any science of biogenesis that claims or demands such a thing is false. It would be a demand that a higher order of things be subject to a lower order, instead of a lower order of things being subject to a higher.

What does an allopathic physician know of Homœopathy, whose knowledge lies within the boundary of inert things? What is his criticism worth in the science of biology when he is only acquainted with chemical and mechanical laws? He has never been in correspondence with the higher laws that all higher life is dependent upon, so all his reasoning and his rationalism are in vain, because what we have to do with life is out of the sphere of rationalism, so his very thoughts breed discord, and that harmony can be restored by a natural law is a thing unknown to him.

What is this turmoil in nature? What is this disturbance in the inner man but nature disquieted or distuned? It is full of discord, and it cannot be restored along the line of opposing forces, but by and through similarly acting forces.

Then let the names of such men as Dunham, Lippe, Hering, and Wells enthuse and inspire us with new efforts to follow, not only in their footsteps, but to look farther into the boundless resources of the law. Much has been revealed, still much is covered by the veil; but if we study carefully the law it will unfold itself to our limited vision in a broader and clearer light. Has not our love for the law a future? Will not right triumph? Is this great temple begun by Hahnemann to remain unfinished? No; we must go on perfecting it from day to day, until we can raise the right hand of fellowship on high and swear allegiance to this law, to this law which was first said by the master to be divine; swear allegiance to our principles as laid down in the *Organon*, which are the fellowship of truth. And may we all in the end voice the words of the poet:

> "One God, one law, one element,
> And one far-off, divine event
> To which the whole creation moves."

